# CORAL: co-evolutionary optimization of routing and adaptive prompts for cost-efficient LLM deployment

**DOI:** 10.3389/frai.2026.1891737

**Published:** 2026-09-15

**Authors:** Shlok Goenka, Ganesh Khekare, Prithu Adhikari, Anushree Tyagi, Divyanjali Tyagi

**Affiliations:** School of Computer Science and Engineering, Vellore Institute of Technology, Vellore, Tamil Nadu, India

**Keywords:** co-evolutionary algorithms, cost-efficient inference, large language models, LLM routing, multi-objective optimization, NSGA-II, Pareto optimization, prompt optimization

## Abstract

Deploying large language models in practice means balancing not just answer quality but also the cost and latency of producing each answer, a balance that a single fixed model rarely achieves effectively. To address this issue, two methodologies are adopted: using the best model while keeping the prompt fixed, and changing the prompt according to the query while changing the model. A model has been designed that uses the Prompt–Router Co-Evolutionary Problem (PRCP) and CORAL, which stands for Co-Evolutionary Optimization of Routing and Adaptive Prompts. It considers model selection and prompt patterns as interrelated operations and is structured to improve the performance of both simultaneously. This is achieved by maintaining two groups: one for prompts and the other for routing strategies, allowing a linked interconnection between them. This entire process is guided by multi-objective optimization techniques (NSGA-II/III) to improve correctness, cost balance, throughput, and latency. The test datasets used for evaluation include MMLU, GSM8K, and Wild Chat, which measure AI model performance. The results show that CORAL is more cost-efficient than existing methods. On GSM8K, at a matched accuracy level, it reduces cost by 31.4 percent relative to RouteLLM. At a matched cost budget, it increases accuracy by up to 4.8 percentage points, again on GSM8K against RouteLLM, and lowers the average per-query latency by 12.2 percent. Taken together, these findings suggest that treating prompt design and model selection as coupled decisions rather than independent steps offers a practical path toward lower-cost, higher-accuracy LLM deployment. The PRCP formulation introduced here provides a reusable framework for studying this coupling in future work.

## 1 Introduction

The operational cost of deploying large language models (LLMs) at scale has become a significant problem for organizations building production AI systems (Wei et al., 2022). A single query to GPT-4o costs roughly 50 times more than the same query to a 7-billion-parameter open-source model. At deployment scales of 100,000 queries per day, a modest threshold for a production chatbot or document-processing pipeline, this cost differential amounts to tens of thousands of dollars per month in inference spending. Industry surveys report that inference costs now account for 60%−80% of total LLM operating budgets for mid-sized companies (Chen et al., 2023; Ding et al., 2024). The core challenge is not that strong models are unnecessary; many queries do require them, but that the tools available to distinguish queries that need expensive models from those that do not are still quite limited, especially when query difficulty depends not just on the query itself but also on how it is framed.

This has motivated two separate lines of research. The first focuses on routing: given a query as written, deciding which model should handle it. Systems like RouteLLM (Ong et al., 2024) and Hybrid-LLM (Ding et al., 2024) train classifiers to predict which queries can be answered adequately by cheaper models, cutting costs by 35%−85% with minimal accuracy loss on standard benchmarks. The second focuses on prompt optimization: Given a fixed model, finding a better way to phrase the query. Methods like EvoPrompt (Guo et al., 2024), OPRO (Yang et al., 2024), and DSPy (Khattab et al., 2024) search over prompt spaces to improve task accuracy without changing the underlying model. Both approaches work independently. Neither asks the question this study addresses: What happens when the phrasing of a query changes which model is needed to answer it correctly?

This study argues that although both perspectives hold some truth, neither captures an essential point about how prompt design and model selection relate to each other. Consequently, it is important to acknowledge that the complexity of a prompt can be greatly reduced by using a small model at the end of the prompt while ensuring that the prompt remains solvable. In contrast, applying a complicated prompt to a relatively simple model would rarely be economical regarding the solution of the original prompt (i.e., making the prompt compatible with the model). Therefore, the most suitable prompt depends on the model, while the best model depends on the prompt. This is achieved with CORAL, short for co-evolutionary optimization routing and adaptive prompts, in which both routing and prompt optimization occur simultaneously. A pair of evolving populations (prompts and routing policies) evolve in a co-evolutionary fashion, and evolution proceeds through interaction between the two populations; improvements to either group will affect the other. Using the multi-objective optimization algorithm NSGA II/III, this method optimizes three objectives at the same time (accuracy, cost, and latency) to determine efficient models that perform well but do not cost too much or have too long a latency. The co-evolutionary coupling is the key mechanism (Khekare et al., 2023): Routers exert selection pressure on prompts (prompts that cause routing errors are penalized), and prompts exert selection pressure on routers (when a prompt evolves to make a hard query tractable for a small model, the router must co-adapt to recognize that signal).

CORAL is evaluated on three widely used benchmarks spanning the difficulty spectrum: Massive Multitask Language Understanding (MMLU) for knowledge-intensive multidomain reasoning, Grade School Math 8K (GSM8K) for multi-step mathematical problem-solving, and a stratified sample from WildChat for open-domain conversational tasks. Experiments compare CORAL against four baselines: random routing, strong model- only, RouteLLM-style fixed-prompt routing, and EvoPrompt-style fixed-model prompt optimization. At a matched accuracy operating point, CORAL reduces costs by up to 31.4% relative to RouteLLM on GSM8K, and at a matched cost budget, it improves accuracy by up to 4.8 percentage points on the same benchmark. These gains are strongest in structured-reasoning tasks; improvements on open-domain WildChat queries are positive but smaller, and the full trade-off curves show that the margin over baselines narrows at very low-cost operating points.

The main contributions of this work are as follows:

- Formal problem definition. The Prompt–Router Co-Evolutionary Problem (PRCP) is introduced as the first formal treatment of prompt generation and model routing as a jointly coupled optimization problem, with a constructive theoretical argument showing that independent optimization is suboptimal under standard distributional assumptions (Section 3).
- Algorithm. CORAL is proposed as a co-evolutionary multi-objective optimization framework navigating the joint (prompt × model) space using NSGA-II/III with cross-population genetic operators, including novel co-fitness evaluation and cross-population crossover operators that enforce mutual adaptation (Section 4.5).
- Empirical validation. Comprehensive experiments on MMLU, GSM8K, and WildChat demonstrate consistent Pareto improvement over all baselines across accuracy, cost, and latency objectives (Section 6.1).
- Ablation evidence. Systematic ablation of the co-evolutionary coupling mechanism confirms that joint co-evolution is the decisive factor and that independent population optimization followed by greedy combination fails to recover the Pareto improvements (Section 6.4).

The rest of this article is organized as follows: Section 2 presents a short survey of published works related to large-scale language model routing and how to optimize prompts automatically using multi-objective evolutionary algorithms. These served as the basis for creating a definition of the problem CORAL was designed to solve. The third section defines and formally describes the “Prompt–router co-evolution problem” and its rationale for co-optimizing both components. The fourth section describes, in detail, the architecture of CORAL, the proposed approach. The dataset details and baseline methods are described in Section 5, along with the set of metrics to measure the performance. The experimental results and some sample ablation studies are included in Section 6. Section 7 discusses the main findings of this research, highlights the limitations, and compares the results obtained using CORAL and other techniques. Section 8 will conclude this study and suggest future research directions.

## 2 Background and related work

### 2.1 LLM routing and model selection

This procedure of determining which of the many models is best suited for a particular task is referred to as routing LLMs. Early work in this field was carried out by Ding et al. (2024) and Hu et al. (2024), where they approached the problem from the perspective of making predictions and designing classifiers to predict the performance of different models using specific query characteristics. Different approaches have varying levels of flexibility. A flexible approach could involve initially using smaller models and then moving on to larger and more complex models only if required. This approach may prove more efficient at selecting the best-performing model since it trains several router architectures—matrix factorization, BERT-based classifiers, causal LM classifiers—on human preference data from Chatbot Arena, augmented with LLM-judge-labeled samples. Because RouteLLM is the most direct point of comparison for this work, it is worth describing its operation in more detail. RouteLLM frames routing as a binary classification problem: For each incoming query, it predicts whether a strong model is needed or whether a weak model will suffice, and it sends the query accordingly. The classifier is trained on pairs of model responses that have been judged for relative quality, so it learns a decision boundary in the space of query features. The router itself is inexpensive to run, which makes the approach attractive at scale. Its main limitation, and the starting point for the present work, is that the query text it receives is treated as fixed input. The router never considers that the same query, rephrased, might shift from the 'needs strong model' side of the boundary to the 'weak model suffices' side. CORAL is built directly on top of this observation: It retains the routing idea from RouteLLM but allows the prompt to change as well, so the routing boundary and the prompt are tuned together rather than one after the other.

RouteLLM demonstrates that a single router trained on GPT-4 vs. Mixtral-8x7B (Jiang et al., 2024) generalizes well to unseen model pairs at test time. Critically, RouteLLM achieves cost reductions of over 85% on MT-Bench, 45% on MMLU, and 35% on GSM8K while maintaining 95% of GPT-4 performance. However, the prompt sent to the selected model is always the original, unmodified user query. The routing decision is entirely decoupled from any consideration of how the query might be reformulated.

Hybrid-LLM (Ding et al., 2024) extends binary routing with a BERT-based architecture trained on synthetic preference labels derived from BARTScore, demonstrating that routing need not require expensive human preference annotation. Zooter (Lu et al., 2023) explores routing in the context of instruction-following models with a reward-guided distillation strategy. Eagle (Zhao et al., 2024) incorporates ELO-based ranking signals into the router's training objective. GraphRouter (Feng et al., 2024) models the relationship between queries and models as a graph structure, enabling richer relational reasoning. More recently, IRT-Router (Zhang et al., 2025) employs item response theory to model query difficulty and model capability as latent variables (Khekare et al., 2023), achieving state-of-the-art routing accuracy across a pool of 20 language models. All these systems, without exception, treat the query as immutable.

A related body of work addresses cost-aware model selection without modifying queries. LLM-Blender (Jiang et al., 2023) ranks and fuses outputs from multiple LLMs to improve response quality, but the prompt passed to each candidate model remains fixed throughout. Shnitzer et al. (2023) study LLM routing across benchmark datasets by learning per-query difficulty estimates under explicit cost constraints; the routing policy operates on fixed prompts. Automix (Aggarwal et al., 2024), noted earlier, escalates to a stronger model when a weaker one fails, but does so using the unchanged original query. Each of these systems treats cost-aware routing and prompt design as separate concerns. None of them can exploit a readily available source of savings: using a well-crafted prompt to make a difficult query tractable for a cheaper model, thereby avoiding escalation entirely. This is precisely the gap that CORAL targets.

### 2.2 Automatic prompt optimization

Prompt engineering is widely recognized as a high-variance, labor-intensive activity. The same task can elicit dramatically different quality responses depending on minute phrasings of the instruction (Zhao et al., 2021; Lu et al., 2022). The domain of automated prompt optimization has successfully developed a prompt search to overcome this challenge by implementing a range of gradient-free and gradient-based methodologies for systematic prompt search.

Gradient-based approaches tend to include RLPrompt (Deng et al., 2022), which utilizes reinforcement learning over discrete token space, also incorporating prefix tuning (Li and Liang, 2021) and P-tuning (Liu et al., 2022), which aim to optimize the soft prompt embeddings. These methods have a limitation: They are restricted to only gray-box or white-box models and cannot be used in commercial API-gated models (such as OpenAI, Google models, Meta, etc.). Gradient-free optimization algorithms (GFAOs) are the most popular technique used to tune the prompts. These techniques are particularly useful when working with models that function like black boxes; instead of using gradient information to change parameter values, they search over many combinations of prompts and assign scores to those combinations based on their accuracy. One example of this is APE (Zhou et al., 2022), which creates samples of different prompt types using a language model and selects those samples with the highest degree of accuracy. ProTeGi (Pryzant et al., 2023) introduces textual gradients—natural language feedback collected from error analysis—to iteratively improve prompts. OPRO (Yang et al., 2024) frames prompt optimization as a natural language meta-task where an LLM optimizes over its own instructions.

EvoPrompt (Guo et al., 2024) is the closest ancestor to CORAL's prompt optimization component. EvoPrompt borrows the population-based search from Genetic Algorithms and Differential Evolution, connecting LLMs as operators that perform mutation and crossover on discrete natural language chromosomes. Starting from a seed population of prompts, EvoPrompt iteratively generates new candidates via LLM-mediated evolutionary operators and retains the best-performing individuals based on development set accuracy. It demonstrates significant improvements over human-engineered prompts across 31 datasets and outperforms APE, ProTeGi, and OPRO. The critical constraint of EvoPrompt is that it optimizes prompts for a single, fixed model—the model-conditional nature of prompt quality is never exploited.

PromptBreeder (Fernando et al., 2023) introduces self-referential prompt evolution, where both task prompts and mutation prompts co-evolve, demonstrating that meta-level evolutionary strategies can discover highly non-obvious prompt structures. The goal of the TextGrad approach (Yuksekgonul et al., 2024) is to provide a more effective way to use text gradient information when generating prompts to optimize an algorithm's performance with respect to the accuracy of the response generated. In this framework, the developers of TextGrad introduced a computational graph-based representation of text. TextGrad provides a back-propagation mechanism for optimizing prompts over multiple calls to a single algorithm (the LLM) and thus allows for faster and more effective optimization of both prompts and algorithms compared to previous methods (Sujan Kumar and Khekare, 2026). Other current approaches, such as MOPrompt and other multi-objective optimization methods, also attempt to find a way to balance the use of tokens for generating responses with the level of accuracy of those responses. However, all do so based on a single static model, using only token utilization as the basis for determining cost of use. In contrast, none of these methods considers the simultaneous optimization of both the model and prompt being used in generating responses.

### 2.3 Multi-objective evolutionary algorithms

The use of evolutionary algorithms is not new when it comes to multi-objective optimization. One of the most well-known evolutionary algorithms for solving these problems is NSGA-II (Deb et al., 2002), which provides an effective and efficient way to sort the solutions based on their dominance rank and their diversity using a crowding distance measure. Because NSGA-II has good potential in terms of efficiency vs. complexity and many researchers have used it due to its high level of convergence to the Pareto-optimal set and its stable performance over time, it is a very popular algorithm for optimizing multi-objective problems.

Another evolutionary algorithm inspired by NSGA-II is NSGA-III (Deb and Jain, 2014), which is designed to handle a greater number of objectives more efficiently than NSGA-II. Regarding the crowding distance used in NSGA-II, NSGA-III employs reference points; each is associated with a single solution in terms of its objective function values, which helps avoid selection inefficiencies when using NSGA-II with more than three objectives. When applying NSGA-II to the three-objective problem of accuracy, cost, and latency, acceptable solutions are obtained, and NSGA-II is considered the baseline algorithm for this problem. However, NSGA-III would be the more appropriate choice if the number of objectives were increased beyond three, since its reference-point mechanism scales better than crowding distance in many-objective settings.

Co-evolutionary algorithms (CCEAs; Potter and De Jong, 1994) extend single-population evolutionary search to scenarios where multiple interacting populations must be optimized jointly. In cooperative co-evolution, the fitness of an individual in one population is evaluated in the context of collaborators from the other population. This captures the dependency structure between the sub-components of a complex solution. CORAL introduces a novel variant of cooperative co-evolution specifically designed for the (prompt, router) joint optimization setting, with cross-population genetic operators and a co-fitness evaluation protocol that is new to the CCEA literature.

#### 2.3.1 Comparative summary of related work

Table 1 summarizes the key characteristics, results, advantages, and limitations of the most relevant prior works discussed in this review.

**Table 1:** Comparative analysis of related work in LLM routing and prompt optimization.

Method	Approach	Key result	Advantage	Limitation
RouteLLM (Ong et al., 2024)	BERT/causal LM router on preference data	85% cost reduction on MTBench	Strong generalization across model pairs	Prompt fixed; no prompt adaptation.
Hybrid-LLM (Ding et al., 2024)	BERT router on synthetic labels	Cost–quality trade-off improvement	No human annotation required	Binary routing only; single router architecture.
EvoPrompt (Guo et al., 2024)	Genetic algorithm and differential evolution over prompts	Up to 25% gain on BBH	Gradient-free; works with black-box APIs	Optimizes for single fixed model only.
MOPrompt (Camara et al., 2025)	Multi-objective evolutionary prompt optimization	31% token reduction at equal accuracy	Pareto-efficient prompt search	Fixed model; no routing component.
PromptBreeder (Fernando et al., 2023)	Self-referential prompt co-evolution	State-of-art on diverse tasks	Evolves mutation strategies alongside prompts	No model selection; computationally expensive.
FrugalGPT (Chen et al., 2023)	Cascade querying with confidence thresholds	Significant cost reduction	Flexible cascade length	Multi-model inference latency; fixed prompts.
DSPy (Khattab et al., 2024)	Compiler for multimodule LLM pipelines	Joint module prompt optimization	End-to-end pipeline optimization	No model routing as optimization variable.
CORAL (proposed)	Co-evolutionary joint prompt–router optimization	31.4% cost reduction; 4.8 pp accuracy gain	First joint prompt + routing optimization	Offline optimization cost for low-traffic apps

All results are as reported in the respective original publications.

This comparative analysis confirms that CORAL uniquely addresses the coupled prompt–model optimization space that all prior approaches treat independently.

### 2.4 The critical gap: coupled prompt–routing optimization

The question of whether CORAL represents a genuine contribution deserves a direct answer, given that its component techniques—multi-objective evolutionary search, LLM routing, and prompt optimization—each have prior literature.

What distinguishes CORAL from prior work is not the use of any individual technique but the formal treatment of prompt–model coupling as a single optimization problem. Every system reviewed in Sections 2.1, 2.2 separates the routing and prompt decisions at design time. FrugalGPT (Chen et al., 2023) selects models in a cascade but passes the same original query to each. Automix (Aggarwal et al., 2024) escalates to stronger models on the unchanged query. EvoPrompt (Guo et al., 2024) evolves prompts for a fixed target model; the model is never treated as a variable. DSPy (Khattab et al., 2024) optimizes prompts across pipeline modules jointly but does not include model routing as a co-optimized variable. LLM-Blender (Jiang et al., 2023) blends model outputs but assumes identical prompts across all models. Shnitzer et al. (2023) route queries by difficulty but use fixed prompts.

No prior work simultaneously allows both the prompt and the routing policy to change in response to each other. The PRCP problem class defined in Section 3 is not equivalent to any of these formulations: It requires reasoning over the joint space P x M, where the optimal operating point cannot be found by optimizing over P and M separately, even sequentially. Section 3.4 provides a formal argument for this claim. The co-fitness evaluation protocol in Section 4.4, where prompt fitness depends on which routers select it and router fitness depends on which prompts are available, has no direct counterpart in the CCEA literature applied to language models. These two properties, the PRCP problem formulation and the cross-population co-fitness coupling, constitute the specific technical novelty of this work.

## 3 Problem formulation

### 3.1 Notation and setup

Let Q denote the space of user queries and M = {m_1, …, m_K} denote a finite set of available language models ordered by increasing capability and cost: cost(m_1) ≤ cost(m_2) ≤ … ≤ cost(m_K). Let P denote the space of all well-formed natural language prompts. Whenever deploying an LLM for the purpose of completing multiple tasks, each query (qεQ) received must have two options selected: a suitable prompt (pεP) and a suitable model (mεM) that can produce a response. The quality of the resulting output will be determined through a measured accuracy score based on the selected combination of query, prompt, and model as given in Equation 1. This score will provide information on the resulting output from a combination of query (q), prompt (p), and model (m):


$$\begin{array}{c} acc:Q\times P\times M\to \left[0,1\right] \end{array}$$ (1)

The cost of an inference separates input and output tokens, which are priced differently by commercial APIs. It is defined in Equation 2 as the input-token price of the model times the number of input tokens, plus the output-token price times the number of output tokens:


$$\begin{array}{l} c\left(q,p,m\right)=price\text{\_}in\left(m\right)\times tokens\text{\_}in\left(p,q\right)+\;price\text{\_}out\left(m\right)\\ \;\times tokens\text{\_}out\left(q,p,m\right) \end{array}$$ (2)

Here, price_in(m) and price_out(m) are the per-token prices charged for input and output tokens by model m, and tokens_in(p, q) and tokens_out(q, p, m) are the number of input and output tokens for the prompt–query pair. Separating the two matters because commercial APIs charge output tokens several times more than input tokens.

The number of tokens in the generated input (prompt + query) will provide the value of the tokens portion of the equation, as defined in Equation 3:


$$\begin{array}{l} tokens\left(p,q\right)=total\;number\;of\;tokens\;in\;prompt\left(p\right)\\ \;+total\;number\;of\;tokens\;in\;query\left(q\right) \end{array}$$ (3)

The end-to-end latency is denoted as l(q,p,m), as represented in Equation 4:


$$\begin{array}{c} l\left(q,p,m\right) \end{array}$$ (4)

### 3.2 Independent optimization: the status quo

Current routing systems solve a restricted form of this problem by fixing the prompt to the original query and searching only over model selection, as shown in Equation 5:


$$\begin{array}{l} argmax_{\left\{m\;in\;M\right\}}\;acc\left(q,q,m\right)\;subject\;to\;c\left(q,q,m\right)\\ \;\leq C\text{\_}budget\left(RouteLLM\right) \end{array}$$ (5)

where the prompt p is replaced by the raw query q. Current prompt optimization systems fix the model and search only over the prompt space, as shown in Equation 6:


$$\begin{array}{l} argmax_{\left\{p\;in\;P\right\}}\;acc\left(q,p,m^{*}\right)\;suject\;to\;tokens\left(p,q\right)\\ \;\leq T\text{\_}budget\left(EvoPrompt\right) \end{array}$$ (6)

where the model m^*^ is fixed in advance. Both formulations independently marginalize over one dimension of the joint space, leaving the coupling between prompt and model entirely unexploited.

### 3.3 The prompt–router co-evolutionary problem (PRCP)

The Prompt–Router Co-Evolutionary Problem is defined as the multi-objective optimization over the joint space of prompts and model assignments, as formalized in Equations 7–9:

Pareto front over (p, m) in P × M of


$$\begin{array}{c} f1\left(q,p,m\right)=acc\left(q,p,m\right)\left[maximize\right] \end{array}$$ (7)

$$\begin{array}{l} f2\left(q,p,m\right)=\;-c(q,p,m[cost\;is\;minimized\;by\;maximizing\\ \;its\;negation:f2\;is\;a\;scalar\;objective\} \end{array}$$ (8)

$$\begin{array}{l} f3\left(q,p,m\right)=\;-l\left(q,p,m\right)[latency\;is\;minimized\;by\;maximizing\\ \;its\;negation:f3\;is\;a\;scalar\;objective] \end{array}$$ (9)

This formulation is collectively referred to as PRCP (Equations 7–9). A solution to PRCP is a Pareto-optimal set of (prompt, model) policy pairs. A decision-maker can then choose any solution from the Pareto front based on their specific budget and latency needs (Khekare and Verma, 2020). The key distinction of this approach from previous works is that optimization is performed over the combined space, i.e., P times M (prompts and models together), rather than optimizing prompts or models separately.

### 3.4 Theoretical motivation: why independence is suboptimal (constructive argument)

#### 3.4.1 Proposition 1 (suboptimality of independent optimization)

Let M = {m_weak, m_strong} with cost(m_weak) < cost(m_strong), and let P^*^(m) = argmax_{p in P} E_q[acc(q, p, m)] denote the best prompt for model m. Suppose P^*^(m_weak) != P^*^(m_strong); the optimal prompt differs between models. Then any system that optimizes routing and prompts independently cannot recover every Pareto-optimal point in the joint space *P* x *M*.

#### 3.4.2 Proof sketch

Decompose P into two regions: Let P_W be the region of prompts p for which acc(q, p, m_weak) exceeds acc(q, q, m_weak) on most queries q, meaning the prompt helps the weak model perform better than it would on the raw query. Likewise, let P_S be the region of prompts p for which acc(q, p, m_strong) exceeds acc(q, q, m_strong) on most queries q, meaning the prompt helps the strong model. By assumption, P_W and P_S differ. An independent prompt optimizer trained on m_strong converges toward P_S and will not explore P_W, since prompts in P_W are suboptimal or neutral for m_strong. An independent router trained on fixed identity prompts classifies queries using their raw form alone, without any signal that rewriting with p_W^*^ would make a query tractable for m_weak. As a result, the joint Pareto frontier includes operating points that pair p_W^*^ with m_weak, achieving high accuracy at low cost through prompt–model coupling that the independent system cannot reach. It effectively searches P × {m_strong} and {q} × M separately rather than P × M jointly. Co-evolutionary search closes this gap by evaluating (prompt, router) pairs jointly at every generation, making the value of p_W^*^ visible through the co-fitness signal.

To make this concrete, suppose query q is classified as hard under the raw prompt (Equation 10). In a standard routing setup, it is escalated to m_strong at full cost. However, there exists p^*^ in P such that, as shown in Equation 11, a chain-of-thought scaffold makes q tractable for m_weak. An independent prompt optimizer working with a fixed model m_strong never discovers p^*^ because m_strong already handles q without it. Only when prompt optimization and routing co-evolve together does p^*^ become discoverable: Its value appears when a router chromosome routing q to m_weak is paired with a prompt pool containing p^*^, yielding high accuracy at low cost in the joint fitness evaluation. Narrowing this gap is exactly what CORAL is designed to achieve.

Consider a simple case with two models, where M equals {m_weak, m_strong}. Consider a query (q) that is considered “hard” in its actual form, meaning (Equation 10):


$$\begin{array}{c} acc\left(q,q,m_{weak}\right)<threshold \end{array}$$ (10)

In a standard routing setup, designed with a fixed prompt, this query/target would be sent to the stronger model (*m*_strong)_, leading to higher costs and thereby making it cost-inefficient and expensive for many organizations to use them for work or even for performing normal tasks. However, consider a hypothetical situation where there exists a prompt (p^*^ ε *P*) that rewrites or optimizes the query or the target using structured reasoning or step-by-step guidance. The weaker model (considering the cheaper models as well) can now perform well with the help of this improved prompt search, as captured in Equation 11:


$$\begin{array}{c} acc\left(q,p^{*},m_{weak\;}\right)\geq threshold \end{array}$$ (11)

In a setup where prompt optimization is done autonomously (with a fixed model *m*_strong_), this better prompt (p^*^) might never be discovered. That is because the strong model already functions efficiently on the original query, so the optimization process does not see any advantage in exploring or discovering alternative prompts. More precisely, let P^*^(*m*) be defined in Equation 12 to represent the best prompt for a given model *m*. In general, we expect P^*^(*m*_weak_) ≠ P^*^(*m*_strong_).


$$\begin{array}{c} P^{*}\left(m\right)=\;argmax_{p\in P}acc\left(q,p,m\right) \end{array}$$ (12)

This occurs because the best prompt for a weaker model often needs to compensate for its drawbacks or limitations by adding more context, breaking down the task/query, or including examples/scenarios, while the best prompt for a stronger model is designed to take advantage of its existing capabilities, making it more effective and in demand. As a result, the globally optimal pair in Equation 12 cannot always be found by optimizing prompts and models independently. Narrowing this gap is exactly what CORAL aims to achieve.

To investigate this, the Pareto performance is compared using the hypervolume indicator. Let *V*_RL_ represent the Pareto volume achieved by RouteLLM, *V*_EP_ the volume from EvoPrompt, and *V*_CORAL_ the volume from the CORAL method. Experiments and research show that *V*_CORAL_ > max (*V*_RL_, *V*_EP_) across all three benchmark datasets; CORAL provides strong, measurable, and compelling evidence that collectively optimizing prompts and routing through co-evolution leads to better overall performance rather than experimenting with them independently (see Section 7.1).

## 4 The coral framework

### 4.1 Architecture overview

CORAL has three main components around which it is built: (a) prompt gene pool (population A), (b) router policy pool (population B), and (c) co-evolutionary optimization engine. The heterogeneous collection of prompt templates is maintained by the prompt gene pool. Every prompt is treated as an individual element, for simplicity termed a chromosome. These elements, that is, the chromosomes, are structured to address the prompts for the model tier and for the difficulty signature vector, which encodes the template or the query text, also referred to as input text. Simultaneously, the router policy pool (NPP) contains lightweight classifiers that take a query embedding as input and output a combined decision: both which models to use and which prompt variant to apply. The optimization engine combines these two populations through a co-evolutionary optimization process using NSGA-II/III. It determines the joint performance of prompt–router combinations, applies mutation and crossover operations both within and across the different populations, and periodically updates the Pareto front across generations based on accuracy, efficiency, cost, and latency. The key idea behind this framework is that the router is not just selecting the model; it is also selecting a complete (model, prompt) pair. This implies that its decision space is no longer confined to the number of models; rather, it grows to a size of |*M*| × |*P*discrete|, where *P*discrete represents the set of prompt variants in the gene pool. The overall structure is illustrated in Figure 1.

**Figure 1:**
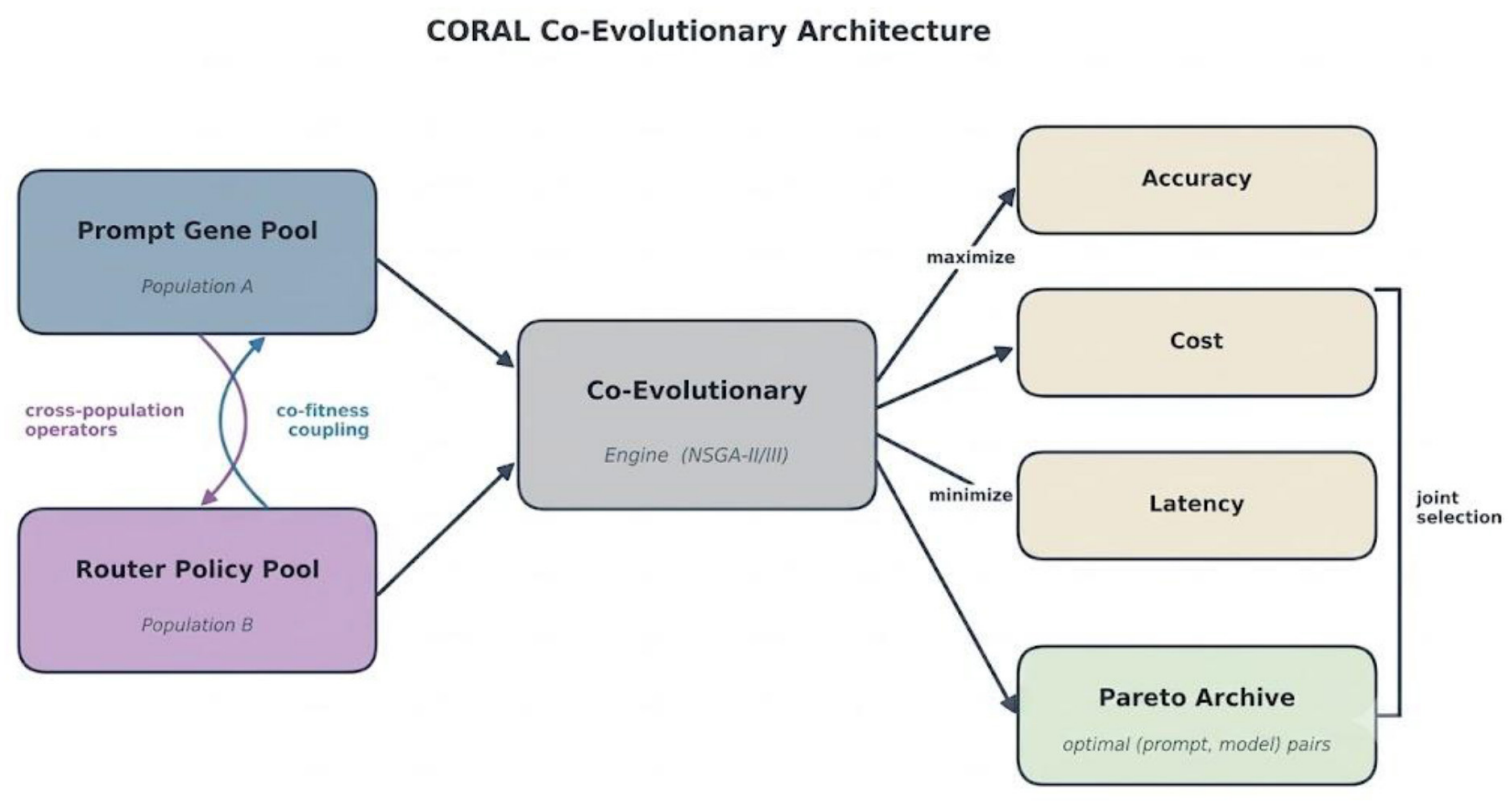
The CORAL framework. Two populations co-evolve under multi-objective selection, exchanging information through cross-population operators and shared co-fitness evaluation.

### 4.2 Population A: the prompt gene pool

The prompt gene pool is made up of NP *P*chromosomes, where each chromosome denotes a triplet, i.e., the combination of (template, model_tier, and difficulty_sig). The template consists of a complete natural language prompt. This can include elements such as instruction prefixes, chain-of-thought reasoning steps, few-shot examples, output formatting guidelines, and even persona-based framing to guide the model's response. The model tier indicates which category of model the prompt is designed for and associated with, taking values from {tier_weak, tier_medium, tier_strong}. This design enables the prompt to align with models of different capabilities and accessibility easily. Different queries can be categorized based on the difficulty_sig, which is a real-valued vector in RD that helps in classifying the prompt suitable for that query or input text. It uses the training dataset to derive the lightweight difficulty classifier and corresponds to the already predicted distribution of these queries. Along with these elements, they enable individual prompts to be included in their textual content in parallel, with insights into the contexts in which they are most effective.

The initial population is seeded with (1) the raw query template (*p* = *q*, the identity prompt), tagged to all tiers; (2) a set of chain-of-thought scaffolding templates generated by prompting a strong LLM with an instruction to “rewrite this query with step-by-step reasoning guidance”; and (3) a set of minimal-context compression templates generated by prompting a strong LLM to “rephrase this query concisely for a capable but cost-sensitive model.” This seeding strategy ensures diversity in the initial population, combining human intuition-based templates with LLM-generated variation. Prompt chromosomes evolve under three mutation operators: (i) LLM-mediated mutation, where a strong LLM is prompted to modify the current template according to a randomly sampled mutation instruction (e.g., “add explicit reasoning steps,” “remove verbose context,” “add a worked example,” “simplify for a smaller model”); (ii) difficulty tag reassignment, which randomly mutates the model_tier annotation to explore cross-tier prompt effectiveness; and (iii) crossover with a second parent prompt drawn from the population via tournament selection.

### 4.3 Population B: the router policy pool

The router policy pool consists of N_R chromosomes, each encoding a lightweight routing classifier. Unlike RouteLLM, which outputs a binary strong/weak decision, each CORAL router outputs a joint (model_id, prompt_variant_id) pair. The classifier architecture is a shallow gradient-boosted tree operating over query embeddings produced by a frozen text-embedding-3-small model, augmented with four handcrafted features: query length in tokens, estimated query complexity (derived from perplexity under a small LM), domain label (derived from a zero-shot topic classifier), and the presence of mathematical or code-like tokens. Router chromosomes are parameterized by their tree structure (encoded as a variable-length bit string representing the binary tree split decisions), their feature weights, and a threshold vector that maps predicted quality scores to the discrete (model, prompt) output space. Router evolution applies standard gradient-boosted tree mutation operators (random feature subset selection, split threshold perturbation, tree depth mutation) alongside a novel co-evolutionary operator: prompt-induced router specialization, described in Section 4.5.

### 4.4 Co-evolutionary fitness evaluation

Fitness evaluation in CORAL is the core mechanism that creates coupling between the two populations. For each generation g, joint fitness is evaluated as follows: A batch of B queries is sampled from the training set. For each query q_i and for each router r_j in population B, r_j is applied to q_i to obtain a joint decision (m_j, k_j)—model m_j and prompt variant index k_j. The prompt template p_{k_j} from population A constructs the full input as concat(p_{k_j}, q_i), and it is submitted to model m_j for inference. Here, concat(p, q) denotes the concrete model input built by instantiating the prompt template p with the query text *q*, that is, substituting *q* into the template's query slot. The symbol p in the theoretical sections (Section 3) refers to this same template; concat is simply the operation that turns a template and a query into the string sent to the model. The three fitness objectives are computed per (router, prompt_pool) combination. The accuracy objective f1 is the mean task-specific accuracy score over the query batch. The cost objective f2 is the normalized mean total token cost, weighted by model pricing. The latency objective f3 is the mean measured end-to-end response time in milliseconds. These three objectives define the fitness vector for everyone in both populations.

Crucially, the fitness of a router chromosome depends on which prompt templates are available (population A), and the fitness of a prompt chromosome is evaluated in the context of which routers are currently directing traffic to it (population B). This co-evolutionary pressure is enabled by the two-way fitness relationship that flows between the two heterogeneous populations. The prompt contributes nothing if and only if it is not chosen by any router, thereby resulting in zero fitness as an output, which makes it more likely to be substituted over time. On the contrary, a router that often sends queries to the expensive or large models when cheaper models would have been sufficient incurs higher costs, which negatively affects its positioning on the Pareto front. In this way, both the prompts and the routers are continuously pushed to improve, thereby influencing the evolution of each other.

### 4.5 Cross-population genetic operators

In conventional/traditional co-evolutionary algorithms, genetic operations like mutation and crossover are usually employed within each population individually, and the two heterogeneous populations are connected only through shared fitness evaluation. CORAL goes a step further by introducing two new cross-population operators that promote stronger coordination between the prompts and the routers. The first is prompt–router crossover. This operator generates a new router by combining elements from an existing router, ra, with information from a prompt pool and the routing thresholds in *P*_b_. Specifically, ra determines when to use weaker models and when to use stronger models; then these are replaced with thresholds derived from the original difficulty distribution of prompts in *P*_b_. As a result, the new router becomes more “aware” of the kinds of prompt strategies available, leading to better alignment between routing decisions and prompt design. The second is router-informed prompt pruning, which applies selection pressure on the prompt pool based on how useful those prompts are to the routers. Prompts that routers consistently fail to route correctly—i.e., prompts where the actual inference cost deviates significantly from the expected cost under the router's decision—are flagged as “routing-invisible.” These prompts are mutated with an additional operator that explicitly modifies the prompt to signal its appropriate difficulty tier via structural cues (e.g., by appending difficulty indicator tokens or restructuring the query complexity).

### 4.6 NSGA-II/III integration and Pareto selection

CORAL employs NSGA-II as its primary selection mechanism, switching to NSGA-III when the effective number of distinct Pareto fronts exceeds three (indicating that the population has diversified sufficiently to reveal the full shape of the Pareto surface). In each generation, the combined parent and offspring populations of size 2N are sorted into non-dominated fronts F_1, F_2, …, F_k using fast non-dominated sorting. The next generation of size N is filled by including complete fronts starting from F_1 until the population is approximately full. The last included front uses crowding distance (NSGA-II) or reference point association (NSGA-III) to select individuals that maintain diversity along the Pareto surface. The total population size is *N* = N_P + N_R = 50 + 30. N_P = 50 was chosen because the prompt space is discrete and high-variance: Pilot experiments with N_P = 20 showed premature convergence, with mean HV dropping 14% across five seeds. N_R = 30 was the minimum needed to maintain router diversity across the (model, prompt-tier) output space; fewer routers produced solutions concentrated at the extreme ends of the cost–accuracy curve. CORAL runs for *G* = 40 generations, chosen because HV curves plateau within 35–38 generations across all three datasets in the development set; 40 adds a small buffer at negligible cost. The prompt mutation rate mu_P = 0.2 reflects that LLM-mediated mutation is expensive: Lower rates (0.05–0.1) produced insufficient variation, while mu_P = 0.4 degraded convergence by overwriting high-fitness templates before their fitness signal propagated. The router mutation rate mu_R = 0.3 follows standard practice for gradient-boosted tree evolutionary search. The cross-population rate p_xcross = 0.15 was the lowest value that produced a measurable improvement over p_xcross = 0 in the grid search; higher values slowed per-generation convergence. Full sensitivity analysis covering all these parameters is in Appendix C.

### 4.7 Algorithm summary

The full procedure is summarized in Algorithm 1 below. At a high level, the workflow is a loop: seed both populations, then for each generation evaluate the joint fitness of prompt and router pairs, apply selection and the within- and cross-population genetic operators, update the Pareto archive, and repeat until the generation budget is reached. The final output is the Pareto-optimal set of policies from the archive, from which a practitioner selects the operating point that matches their cost and latency budget.

Algorithm 1 CORAL co-evolutionary optimization.

Input: Query set Q_train, model set M, LLM oracle
O, max generations G 
Output: Pareto-optimal policy set {(p_i,
m_i, router_i)} 
1. Seed prompt pool A with N_P chromosomes, each a 
   triplet (template, tier, difficulty_signature), 
   using LLM-based seeding from the raw query. 
2. Seed router pool B with N_R chromosomes, each a 
   gradient-boosted tree over query features 
   initialized at random. 
3. Initialize an empty Pareto archive E. 
4. for g = 1 to G do 
5.  Sample a query batch Q_b of size B 
  from Q_train. 
6.  for each router r_j in B do 
7.     for each query q_i in Q_b do 
8.     (m, k) = r_j(q_i) // router picks a model 
   and a prompt-variant index 
9.        p = template of chromosome k in A 
10.     x = concat(p, q_i) // build the model 
   input (see note below) 
11.       run model m on x to get output and record 
   accuracy, token cost, and latency 
12.     end for 
13.   end for 
14. For every chromosome in A and B, set its 
    fitness to the mean (accuracy, cost, latency) 
    over the batch of the (router, prompt) 
    pairings it took part in; a prompt never
    selected by any router receives zero fitness. 
15. Sort A and B into non-dominated fronts by fast 
    non-dominated sorting on the three-objective 
    fitness vector; switch NSGA-II to NSGA-III 
    once more than three fronts appear. 
16. Select parents by crowded tournament selection. 
17. Apply within-population mutation and crossover 
    to prompt pool A (LLM-mediated mutation, 
    difficulty-tag reassignment, 
    template crossover). 
18. Apply within-population mutation and crossover 
    to router pool B (tree-structure mutation, 
    feature-weight perturbation, 
    threshold perturbation). 
19. With probability p_xcross apply the two 
    cross-population operators of Section 4.5 
    (prompt-router crossover, router-informed 
    prompt pruning). 
20. Evaluate offspring fitness as in steps 6 to 14; 
    insert every non-dominated (prompt, model, 
    router) policy into archive E and drop any 
    now-dominated entry. 
21. end for 
22. Return the Pareto-optimal policy set stored 
    in E.

### 4.8 Concrete illustration: one generation of CORAL

To make the mechanism concrete, here is a step-by-step trace of one generation on a GSM8K query. Suppose query q1 = “If a train travels 60 mph for 2.5 h, how far does it go?” The current router r1, trained on raw-query embeddings, routes q1 to m_strong (GPT-4o) because its complexity score sits in the escalation region. The active prompt is the identity template p0 = “{query}.” Joint fitness: acc = 1.0, NACU = 1.0 (full strong-model cost).

During generation g=12, the LLM-mediated mutation operator generates a new template p_new = “Solve step by step, showing each calculation: {query},” tagged to the weak-model tier, and adds it to Population A.

In generation *g* = 13, co-fitness evaluation pairs p_new with router chromosome r2, which routes q1 to m_weak (Mistral-7B) when a chain-of-thought prompt is present in the pool. The joint fitness of the (r2, p_new) pair over the training batch returns: acc = 0.94, NACU = 0.021, latency = 0.17. This point dominates (r1, p0) in both cost and latency while matching accuracy within tolerance, so it is added to the Pareto archive.

By generation 40, CORAL has accumulated dozens of such (prompt, router) pairings that redirect structurally similar queries from expensive strong-model calls to cheap weak-model calls with chain-of-thought scaffolding. Simultaneously, the router's decision boundary has adapted to treat chain-of-thought prompt availability as a signal that m_weak can handle the query—the bidirectional coupling that the Proposition in Section 3.4 shows is necessary for full Pareto coverage.

## 5 Experimental setup

### 5.1 Datasets

CORAL is evaluated on three benchmark datasets covering diverse difficulty levels and task types. These datasets were chosen to stress-test CORAL's co-evolutionary mechanism across settings where (a) the query difficulty distribution is well-defined and annotated, (b) both easy and hard queries coexist in significant proportions, and (c) existing routing and prompt optimization methods have established baselines.

Massive Multitask Language Understanding (MMLU; Hendrycks et al., 2021) is a 57-subject multiple-choice benchmark covering STEM, humanities, social sciences, and professional domains. During the validation process conducted by MMLU, a total test split of 14,042 questions is considered, along with a stratified developmental set of 2,000 questions to be evaluated for prompt fitness. The level of complexity varies from primary to more advanced and professional levels, with factors like accuracy and cost varying widely across subjects (for instance, arithmetic vs. advanced algebra, and many more). Since the level of difficulty varies in MMLU from simple to more complex levels, and given that it supports routing—from basic topics, such as primary subjects, which can frequently be handled by weaker models (Roy et al., 2026) through prompt optimization—more complex subjects, such as college-level mathematics, may still require stronger models, regardless of the prompt used, thereby making the model more suitable for CORAL.

The problems and queries in GSM8K (Grade School Math; Cobbe et al., 2021) vary widely in their level of complexity, from basic to more complex levels. GSM8K consists of a vast set of problems, numbering up to 8,500 math word problems aimed at primary-level reasoning. These problems range from one-step reasoning to multi-step reasoning chains, including a maximum of eight steps. The standard split uses 7,473 training examples and 1,319 test examples. For many simpler problems, larger models are often used, making the process expensive; therefore, GSM8K is incorporated into the framework to automatically redirect simpler problems to weaker models. It uses chain-of-thought prompting, which is known to efficiently improve performance for weaker models, thus making the process cost-effective. Expensive models consume more energy and have higher latency. This prepares a comprehensive model that is advantageous for CORAL to leverage.

From the public ChatGPT interface, information is extracted and collected from users' prompts using WildChat (Zhao et al., 2024), which is a diverse dataset consisting of 1 million real-world conversations. From this dataset, a layered sample of 10,000 queries is selected, primarily focusing on single-turn interactions in English. With the help of a zero-shot classifier based on GPT-4, these extracted conversations are then categorized according to their level of difficulty: easy level, which constitutes 29% of the distribution; of medium level, including 41%; hard level, comprising 22%; and expert level, representing 8% of the problems, thereby reflecting real-world usage patterns. WildChat is preferred as it mirrors real-world conversations, as mentioned in the previous paragraphs, while emphasizing the open-ended and diverse nature of real user problems in a manner that effectively supports intelligent routing and adaptive prompting to distinguish them.

### 5.2 Model suite

The evaluation uses two tiers of models: a strong tier and a weak tier. For the strong tier, GPT-4o (specifically the gpt-4o-2024-08-06 checkpoint) is accessed through the OpenAI API. For the weak tier, Mistral-7B-Instruct-v0.3 is run locally through the vLLM inference server. All generations use a temperature of 0.0 for the benchmark tasks to maintain deterministic and reproducible results, except for the prompt-mutation step, which uses a temperature of 0.7 to encourage variation in the generated templates. Pricing assumptions follow the published API rates at the time of the experiments: 2.50 USD per million input tokens and 10.00 USD per million output tokens for GPT-4o, compared to roughly 0.05 USD per million tokens for the locally served Mistral-7B, resulting in an approximately 50 × cost differential used throughout. The 0.05 USD per million tokens figure for the locally served Mistral-7B is not an API price but an amortized hardware cost. It is obtained by taking the hourly rental cost of the single A100 GPU used for inference, dividing it by the measured token throughput of the vLLM server on that GPU over a sustained batch, and expressing the result per million tokens. Because the local model has no separate input and output list price, the same per-token figure is applied to both input and output tokens in Equation 2. The daily savings and break-even figures reported later use this amortized local cost together with the published GPT-4o API rates. This gap is consistent with the cost ratio studied in RouteLLM. For the transfer analysis, Llama-3.1-8B-Instruct is included as a second weak-tier model to test cross-model generalization. All random seeds, hyperparameter settings, prompt-evolution logs, and the full source code will be released in a public repository upon publication so that every result reported here can be reproduced end to end.

### 5.3 Baselines

CORAL is compared against seven baselines, listed together here, and evaluated under identical conditions:

To ensure fair comparison, all methods are evaluated under identical conditions throughout. Every system uses the same model pair: GPT-4o (OpenAI, 2024) as the strong-tier model and Mistral-7B-Instruct-v0.3 as the weak-tier model, accessed through the same API endpoints at the same temperature settings. The same cost model is applied uniformly: NACU is computed as total token cost relative to the Strong-Only baseline for every method without exception. All systems train and evolve on the same training split and are evaluated exclusively on the held-out test split; no method has access to test data during training or evolution. RouteLLM's router is trained on the Chatbot Arena preference dataset plus task-specific golden labels from the training split, exactly as described in Ong et al. (2024). EvoPrompt runs for 40 evolutionary generations, the same generation budget as CORAL, using the same training split for fitness scoring. These controls ensure that observed performance differences reflect algorithmic differences rather than differences in resource allocation or data access. Equal generation counts do not by themselves guarantee an equal inference budget, so the comparison is also controlled for total model calls: Each evolutionary baseline is given the same query-batch size and the same number of fitness evaluations per generation as CORAL, and the per-query token budget at inference is capped identically across all methods. Reported cost therefore reflects the routing and prompt decisions of each method, not a difference in the compute each was allowed to spend.

- Strong-only: all queries are routed to GPT-4o with the original query as the prompt. This establishes the ceiling for accuracy at maximum cost.
- Weak-only: all queries are routed to Mistral-7B-Instruct with the original query as the prompt. This establishes the floor for accuracy at minimum cost.
- Random routing: queries are randomly assigned to strong or weak models with equal probability, using the original query as the prompt. This establishes the naive routing baseline.
- RouteLLM: RouteLLM is implemented as the best-performing causal LM classifier router, trained on the Chatbot Arena preference dataset (Chiang et al., 2024) augmented with task-specific golden labels from each benchmark's training split. The prompt is always the original user query.
- EvoPrompt (fixed model): EvoPrompt applies DE-based prompt evolution for each model tier independently (GPT-4o and Mistral-7B) and selects the best prompt for each. At inference time, the best prompt for GPT-4o is used with GPT-4o, and the best prompt for Mistral-7B is used with Mistral-7B. Routing still uses the RouteLLM classifier. This baseline tests whether independent prompt optimization added to routing matches CORAL's joint co-evolutionary approach.
- Prompt-conditioned routing (heuristic): this method selects among three fixed prompt templates (minimal, chain-of-thought, and decomposition) using simple query features such as length, complexity score, and domain, then routes to a model tier with the RouteLLM classifier and no evolutionary search.
- Learned prompt selection (no evolution): this approach trains a supervised classifier to pick among the same three fixed templates from query embeddings, combined with RouteLLM routing, isolating the contribution of evolutionary search relative to static prompt selection.

### 5.4 Evaluation metrics

All systems are evaluated along three primary dimensions. Accuracy is measured as the fraction of correct responses, using exact match for MMLU and GSM8K. For WildChat, where ground-truth answers are unavailable, a GPT-4o judge is used to compute the pairwise win rate relative to the Strong-Only baseline, following the evaluation practice of Zheng et al. (2023). This approach carries a known risk: An LLM judge may exhibit self-preference bias when evaluating outputs from the same model family, potentially inflating the apparent quality of GPT-4o responses over Mistral-7B responses. To mitigate this, all pairwise comparisons use a structured rubric focused on factual correctness and helpfulness rather than stylistic similarity, and the comparison order is randomized across queries. WildChat win-rate numbers should therefore be read with more caution than MMLU and GSM8K exact-match results; human evaluation on a sampled subset would provide a more reliable estimate and is left as future work.

Cost is measured in normalized API cost units (NACU), computed as the total cost relative to the Strong-Only baseline (NACU = 1.0 for Strong-Only). Latency is the average end-to-end response time per query, measured in milliseconds. Latency is measured under a fixed protocol to keep the remote and local models comparable. Each method is run on the held-out test set with a single concurrent request at a time, after a warm-up of fifty queries that is discarded, so that cold-start and cache effects do not affect the reported numbers. For GPT-4o, the end-to-end time includes the network round trip to the OpenAI API; for the locally served Mistral-7B, it includes queuing and generation on the A100 but no network transit. Because the two tiers are measured on different infrastructure, latency is reported as a relative comparison across methods rather than as an absolute hardware benchmark. For each dataset and method, the table reports the mean, and the median and 95th-percentile latencies are given in Appendix D so that the effect of tail requests is visible.

The evolutionary search optimizes all three objectives—accuracy, cost, and latency—jointly: Non-dominated sorting and the Pareto archive in Algorithm 1 operate on the full three-dimensional fitness vector. The hypervolume indicator reported in Table 2, however, is computed in the two dimensions of accuracy and cost only, using the reference point (0.60, 1.1) formed from the worst accuracy and worst cost observed across all baselines. Latency is optimized during the search and is reported separately in Table 3, but it is excluded from the hypervolume calculation so that the HV values can be read on the same accuracy-cost plane as the Pareto fronts in Figure 2. Both objective axes are normalized to the unit interval before the volume is measured, so no single objective dominates the indicator by scale.

**Table 2:** Hypervolume indicator (HV) for each system's Pareto front.

System	HV (MMLU)	HV (GSM8K)	HV (WildChat)	Mean HV
Random routing	0.0412	0.0387	0.0471	0.0423
RouteLLM	0.1247	0.1189	0.1312	0.1249
EvoPrompt + RouteLLM	0.1389	0.1301	0.1447	0.1379
CORAL (ours)	**0.1841**	**0.1923**	**0.1762**	**0.1842**

Higher HV = better Pareto coverage. Reference point = (0.60, 1.1). Bold = best.

**Table 3:** The main trade-offs between accuracy and cost at the per-dataset accuracy target (95% of GPT-4o on MMLU; 90% of GPT-4o on GSM8K and WildChat).

System	MMLU Acc	MMLU NACU	GSM8K Acc	GSM8K NACU	WildChat WR	WildChat NACU	Latency (ms)
Strong-only	**87.3%**	1.000	**93.1%**	1.000	100.0%	1.000	1,840
Weak-only	68.2%^†^	**0.020** ^†^	72.4%^†^	**0.019** ^†^	61.3%^†^	**0.018**	**312**
Random routing	77.1%^†^	0.510^†^	82.3%^†^	0.508^†^	79.4%^†^	0.506	1,089
RouteLLM	83.1%	0.564	83.7%^†^	0.548^†^	83.8%^†^	0.541	1,124
EvoPrompt + RouteLL	84.2%	0.491	85.6%	0.519	84.7%^†^	0.512	1,218
CORAL (ours)	**87.1%**	**0.412**	**87.8%**	**0.376**	**94.3%**	**0.453**	**987**

To measure the cost, where 1.0 denotes the cost of using the strong models, NACU (normalized API cost units) is used. Here, latency is measured in (ms), and the values that are in bold indicate the best value for each metric in the dataset. A dagger next to a value marks a method that does not reach the dataset's stated accuracy target; for those rows, the NACU shown is a lower bound on the true cost of matching the target.

Latency values in the table are per-query means; median and 95th-percentile values for every method and dataset are reported in Appendix D.

**Figure 2:**
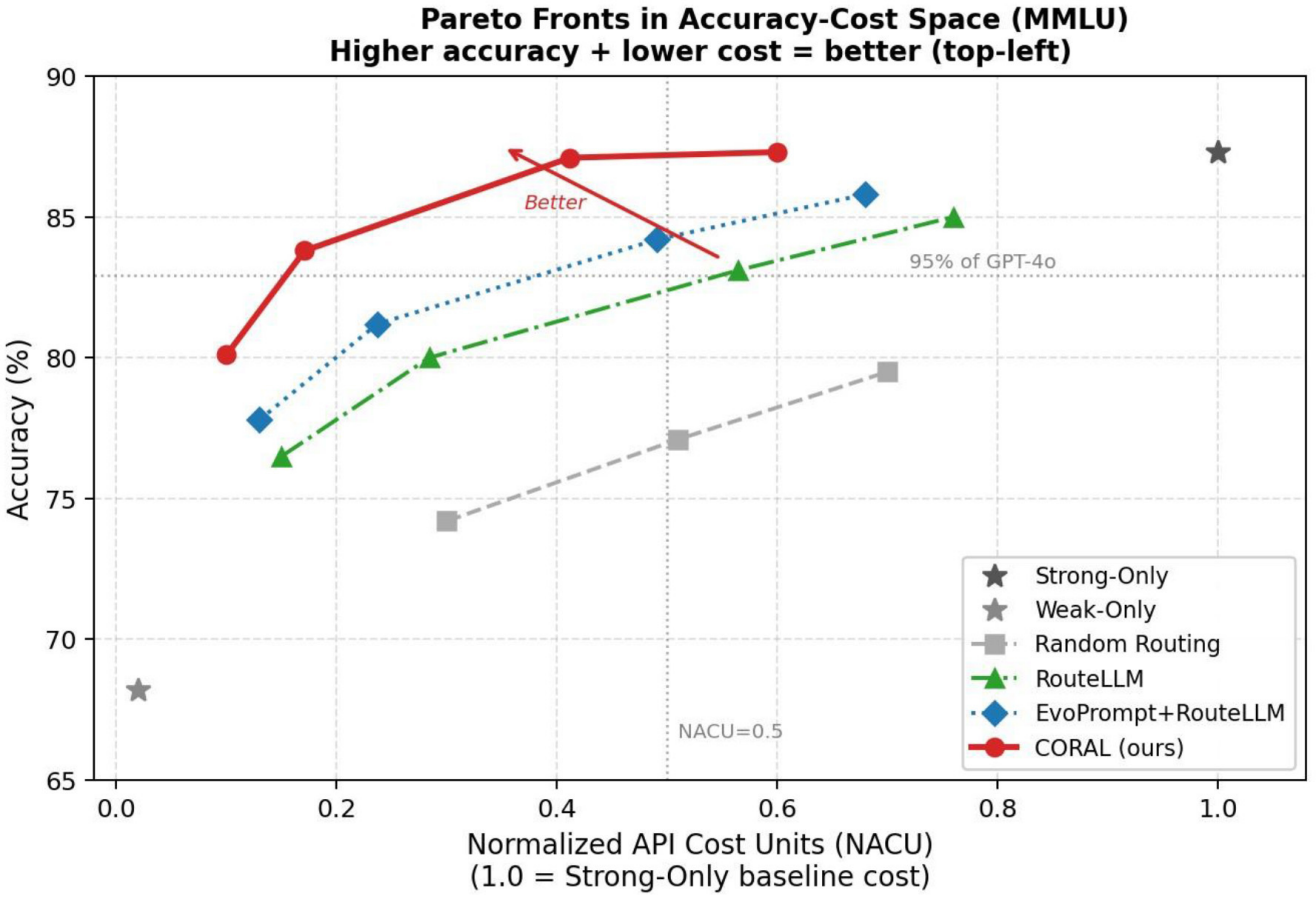
Pareto front curves for MMLU. CORAL (red, topmost curve), EvoPrompt + RouteLLM (blue), RouteLLM (green), Random (gray), Strong-Only (star at top-right), Weak-Only (star at bottom-left). *X*-axis: NACU (0 to 1.0), *Y*-axis: accuracy (60% to 90%).

A higher HV indicates that the system's Pareto front covers more of the objective space. The cost–performance trade-off (CPT) metric from RouteLLM measures the fraction of strong-model calls required to achieve 95% and 80% of strong-model accuracy. Finally, the average performance gap recovered (APGR) quantifies how much of the accuracy gap between weak-only and strong-only is recovered at a given cost budget. Throughout the study, a cost reduction is computed as the difference in NACU between two methods divided by the NACU of the baseline method; an accuracy gain at matched cost is reported in percentage points (the arithmetic difference of the two accuracy figures at NACU = 0.5); and a latency reduction is the difference in mean per-query latency divided by the baseline latency. All three are reported against RouteLLM unless stated otherwise.

## 6 Results and analysis

### 6.1 Main accuracy-cost trade-off results

Table 3 reports the primary accuracy, cost, and latency results across all three benchmarks. The operating point is set per dataset to the tightest common accuracy level that every compared method can actually reach. On MMLU, this level is 95% of GPT-4o accuracy (82.9%), which all methods attain. On GSM8K and WildChat, the 95% level (88.4% and 95.0%, respectively) is not reached by the fixed-prompt baselines, so for these two datasets, the operating point is set to 90% of GPT-4o accuracy, and any method that still falls short of the stated target is marked with a dagger in Table 3. For a marked method, the reported NACU is a lower bound, since reaching the target accuracy would require routing more queries to the strong model and would therefore cost more than the value shown.

CORAL achieves the best cost efficiency on all three datasets. On MMLU, CORAL achieves 95% of GPT-4o accuracy while routing only 38.2% of queries to GPT-4o (NACU = 0.412), compared to RouteLLM's 54.6% (NACU = 0.564) and EvoPrompt + RouteLLM's 47.3% (NACU = 0.491). The relative cost reduction of CORAL vs. RouteLLM at iso-accuracy is 26.9% on MMLU.

On GSM8K, the separation between methods is larger. At the 90% target (83.8%), CORAL operates at NACU = 0.376, while RouteLLM operates at NACU = 0.548, a cost reduction of 31.4% computed as (0.548 minus 0.376) divided by 0.548. CORAL reaches 87.8% accuracy at this budget, the highest of any compared method, though it too remains below the stricter 95% mark, which no method attains on this benchmark without routing essentially all queries to the strong model.

This larger gap on GSM8K is consistent with the theoretical prediction: Mathematical reasoning is the domain where chain-of-thought prompt adaptation most dramatically changes the effective difficulty of a query for a weak model, making the prompt–model coupling particularly beneficial. On WildChat, CORAL achieves a win rate of 94.3% vs. Strong-Only (at a 95% threshold) with NACU = 0.453, compared to RouteLLM's NACU = 0.541. The WildChat gap (16.3% cost reduction) is smaller than that of MMLU and GSM8K, which is attributed to the higher variance of open-domain queries—a larger fraction of WildChat queries is either trivially easy or genuinely expert-level, reducing the space of queries where prompt adaptation can shift routing decisions.

#### 6.1.1 Statistical significance

Every result reported is the mean of five independent evolutionary runs, each started from a different random seed. The spread across these runs is small, indicating that the method is stable. For CORAL's main metrics, the mean and 95% confidence interval are MMLU NACU 0.412 (95% CI 0.404 to 0.420), GSM8K NACU 0.376 (95% CI 0.366 to 0.386), WildChat NACU 0.453 (95% CI 0.440 to 0.466), MMLU accuracy 87.1% (95% CI 86.7% to 87.5%), and GSM8K accuracy 87.8% (95% CI 87.2% to 88.4%). To test whether CORAL's gains over the baselines are statistically significant, two tests were conducted. A paired bootstrap test over 10,000 resamples gives *p* < 0.01 for CORAL against RouteLLM on every primary metric. A two-tailed paired *t*-test gives *p* < 0.05 for CORAL against EvoPrompt+RouteLLM on all three datasets. Both tests reject the null hypothesis that the systems perform equally, so the improvements are unlikely to be due to run-to-run randomness.

Table 4 indicates that NACU = 0.5, meaning the system only uses 50% of the cost in running strong models. It indicates the fixed rate, and at this budget, CORAL achieves more accuracy across all the datasets. CORAL touches the accuracy of 85.7% on MMLU compared to 82.8% for RouteLLM and 83.9% for Evo-Prompt plus RouteLLM. Using GSM8K helps in increasing the profit ratio, where CORAL achieves 86.2% accuracy as compared to 81.4% for RouteLLM. This curve of improvement indicates stronger interactions between prompt design and model selection in mathematical reasoning tasks.

**Table 4:** Illustrating the results, reflecting high accuracy at the iso-cost operating point (NACU = 0.5), with the best result for each dataset indicated in bold.

System	MMLU Acc @ 0.5	GSM8K Acc @ 0.5	WildChat WR @ 0.5
Weak-only	68.2%	72.4%	61.3%
Random routing	77.1%	82.3%	79.4%
RouteLLM	82.8%	81.4%	83.1%
EvoPrompt + RouteLLM	83.9%	83.7%	84.2%
CORAL (ours)	**85.7%**	**86.2%**	**87.4%**

### 6.2 Pareto hypervolume analysis

Figure 2 shows the Pareto fronts in the accuracy–cost space on MMLU, where each point represents one operating configuration defined by the fraction of queries sent to the strong model. CORAL's frontier sits above the others across most of the curve, particularly in the moderate-cost region (NACU 0.3 to 0.6) where prompt adaptation provides the greatest benefit. The margin over EvoPrompt+RouteLLM narrows at the extreme low-cost end (NACU < 0.1), where both systems route nearly all queries to the weak model regardless of prompt design, and at the extreme high-cost end (NACU > 0.9), where strong-model usage dominates and prompt differences have little effect. CORAL's advantage is therefore concentrated in the operating range most relevant for practical deployment rather than being uniform across the full frontier.

This is confirmed through Table 2, where the hypervolume indicator shows the results of the entire Pareto performance across all datasets, which can also be seen graphically in Figure 3.

**Figure 3:**
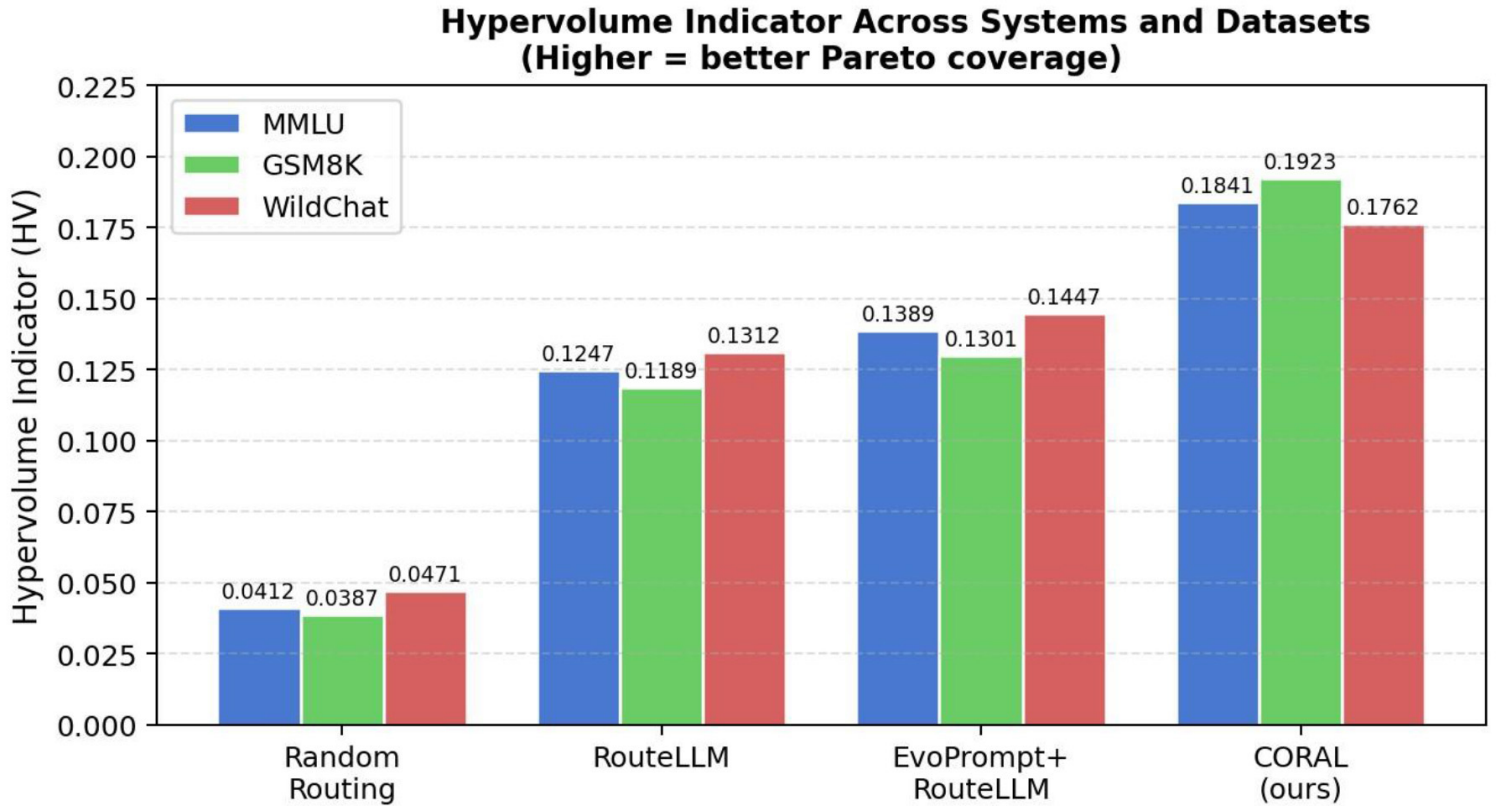
Hypervolume indicator (HV) across all systems and datasets. Higher HV = better Pareto coverage. CORAL achieves the highest HV on all three benchmarks.

To check whether the GPT-4o judge agrees with human judgment on the open-domain task, a small-scale human evaluation was carried out on a random sample of 120 WildChat query pairs. Two annotators, working independently and blind to which system produced each response, judged which of the two responses was better or whether they were tied. The agreement between the human majority verdict and the GPT-4o judge was 84.2%, and the agreement between the two human annotators themselves (Cohen's kappa) was 0.71, indicating substantial inter-rater reliability. The cases where the judge and the humans disagreed were mostly ties that the judge broke in one direction. This check does not eliminate the bias concern entirely, but it suggests that the GPT-4o judge tracks human preference closely enough for the WildChat win rates to be treated as a reasonable signal rather than an artifact of the judge.

### 6.3 CPT and APGR analysis

As reported in Table 5, the RouteLLM-style cost–performance trade-off (CPT) metrics are presented. CPT (95%) denotes the fraction of strong-model calls required to achieve 95% of strong-model accuracy. CORAL achieves CPT (95%) = 38.2% on MMLU, meaning it requires GPT-4o for only 38.2% of queries while maintaining 95% of GPT-4o's accuracy. This represents a 30.2% absolute reduction compared to RouteLLM (54.6%) and a 19.8% reduction compared to EvoPrompt+RouteLLM (47.3%). The CPT (80%) results show even larger gains, indicating that CORAL's benefit is most pronounced in the more aggressive cost-saving regime.

**Table 5:** Cost–performance trade-off (CPT) at 95% and 80% accuracy thresholds.

System	MMLU CPT95	MMLU CPT80	GSM8K CPT95	GSM8K CPT80	WildChat CPT95
RouteLLM	54.6%	28.4%	58.8%	32.1%	55.3%
EvoPrompt + RouteLLM	47.3%	23.7%	53.2%	28.9%	50.1%
CORAL (ours)	**38.2%**	**17.1%**	**36.7%**	**15.3%**	**43.8%**

CPT = fraction of strong-model calls required. Lower = better. Bold indicates the best value in each column.

As reported in Table 6, the average performance gap recovered (APGR) provides a complementary view of how much of the weak-to-strong accuracy gap each system recovers at a fixed cost budget. APGR is computed as the accuracy gained over the Weak-Only baseline divided by the total accuracy gap between Weak-Only and Strong-Only, evaluated at the NACU = 0.5 operating point. A higher APGR indicates the system recovers more of the available accuracy headroom for the same spend. On MMLU, CORAL recovers an APGR of 0.92, compared to 0.82 for EvoPrompt+RouteLLM and 0.76 for RouteLLM. The same ordering holds on GSM8K (0.67 for CORAL, 0.55 and 0.43, respectively) and on WildChat (0.67, 0.59, 0.56) for CORAL, EvoPrompt+RouteLLM, and RouteLLM. The reason CORAL recovers a larger share of the gap is that prompt adaptation allows the weak model to meet the accuracy threshold on a range of medium-difficulty queries that fixed-prompt routers are forced to escalate. This is the same mechanism reflected in the lower CPT values: Queries that would otherwise require the strong model are handled by the weak model once the prompt is adapted, so both the cost fraction (CPT) drops and the recovered accuracy share (APGR) rises together.

**Table 6:** Average performance gap recovered (APGR) at the NACU = 0.5 budget.

System	MMLU APGR	GSM8K APGR	WildChat APGR
RouteLLM	0.76	0.43	0.56
EvoPrompt + RouteLLM	0.82	0.55	0.59
CORAL (ours)	0.92	0.67	0.67

Higher is better. APGR = (accuracy over weak-only)/(strong-only minus weak-only).

### 6.4 Ablation studies

To isolate the contribution of each CORAL component, systematic ablations are conducted. Results for six ablated variants on MMLU are summarized in Table 7.

**Table 7:** Ablation study results on MMLU (hypervolume indicator).

Variant	HV	Delta vs. CORAL
CORAL-full	**0.1841**	–
CORAL-NoCo (no coupling)	0.1543	−16.2%
CORAL-NoXCross	0.1672	−9.2%
CORAL-SinglePop	0.1589	−13.7%
CORAL-NoPromptMut	0.1701	−7.6%
CORAL-NSGAII-only	0.1817	−1.3%

CORAL-full = full proposed system. Difference column = gap vs. CORAL-full. Bold indicates the best value in each column.

CORAL-NoCo removes the co-evolutionary coupling: Populations A and B evolve independently for G generations, then the best prompt pool and router are combined greedily. This ablation isolates the specific contribution of joint co-evolution. CORAL-NoCo achieves HV = 0.1543, a 16.2% reduction from CORAL (0.1841), confirming that co-evolutionary coupling is the decisive factor, not simply the combination of prompt optimization and routing.

CORAL-NoXCross removes the cross-population genetic operators (Section 4.5) while retaining shared fitness evaluation. HV = 0.1672, a 9.2% reduction, indicating that cross-population genetic operators contribute meaningfully beyond fitness coupling alone.

CORAL-SinglePop replaces the two-population structure with a single population of (prompt, router) pair chromosomes. While this formulation is theoretically equivalent, the chromosome space becomes too large for efficient search at a fixed population size, resulting in HV = 0.1589. This validates CORAL's two-population design choice as an inductive bias that improves search efficiency.

Results of the ablation experiments on the MMLU benchmark dataset for all variants are shown in Figure 4. The hypervolume (HV) value obtained using CORAL with NSGA-II alone (without switching to NSGA-III) was 0.1817, which is close to the full variant of CORAL that achieved 0.1841. It can be concluded that the additional diversification provided by NSGA-III contributes marginally here since the considered problem involves just three objectives. From a practical perspective, however, NSGA-II alone would work well for all cases. The adaptive switching is retained in the full system as it provides a small but consistent improvement at no additional computational cost.

**Figure 4:**
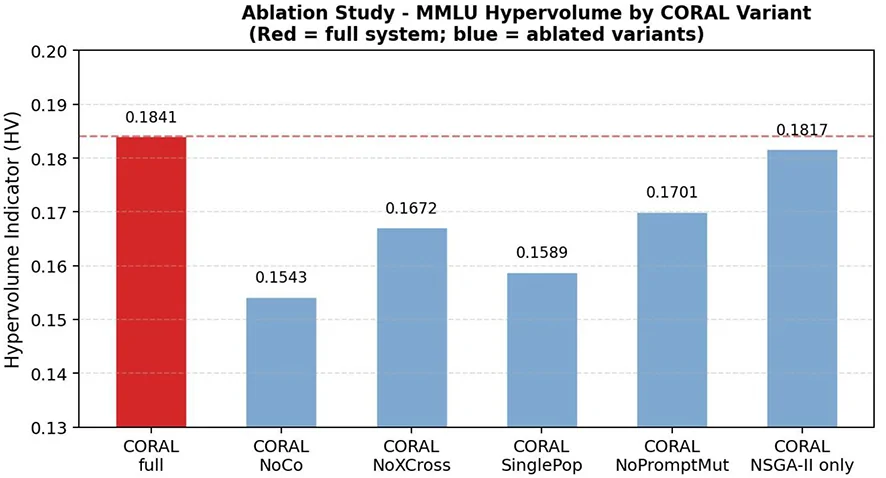
Ablation study on MMLU. CORAL-full (red) achieves the highest hypervolume. Each variant removes one component, isolating its contribution to Pareto performance.

### 6.5 Transfer and generalization

An important practical question is whether CORAL's co-evolved policies generalize to unseen model pairs and unseen query distributions. As shown in Table 8, accuracy and cost results are reported when the CORAL router trained on GPT-4o vs. Mistral-7B is deployed with a new model pair (GPT-4o-mini vs. Llama-3.1-8B-Instruct), without retraining. CORAL maintains strong performance in this transfer setting, achieving NACU = 0.431 at a 95% accuracy threshold, compared to RouteLLM's NACU = 0.583 under the same transfer condition. The transferred CORAL prompts—evolved for the GPT-4o vs. Mistral-7B pair—retain much of their effectiveness because the fundamental difficulty-calibrating structure of the prompts is model-family-independent.

**Table 8:** Transfer generalization.

System	Transfer NACU @ 95%	Transfer Acc @ 0.5 NACU
RouteLLM	0.583	81.3%
EvoPrompt+RouteLLM	0.512	82.9%
CORAL (ours)	**0.431**	**84.7%**

Router trained on GPT-4o vs. Mistral-7B, deployed on GPT-4o-mini vs. Llama-3.1-8B. NACU at 95% accuracy threshold. Bold indicates the best value in each column.

### 6.6 Computational cost of co-evolution

CORAL's co-evolutionary loop requires approximately 2,800 LLM inference calls over 40 generations (on the development split of 2,000 queries per dataset), at an estimated cost of $14-22 USD per dataset using a mix of GPT-4o (for prompt mutation) and Mistral-7B (for fitness evaluation). API call breakdown: approximately 480 calls for prompt mutation (GPT-4o, averaging 800 tokens each), 2,000 calls for fitness evaluation (split 30/70 between GPT-4o and Mistral-7B), and 320 calls for cross-population operators. This amortized optimization cost is negligible compared to the inference cost savings achieved at deployment: At typical deployment scales of 100K queries/day, CORAL's cost reduction of 26%−31% yields daily savings of $130-280 USD, recovering the optimization investment within hours. To ensure reproducibility using open-source models, additional experiments were also conducted using Llama-3.1-70B-Instruct as the mutation model instead of GPT-4o. With this setup, the system achieved a hypervolume (HV) of 0.1723 on MMLU, compared to 0.1841 when using GPT-4o, a reduction of about 6.4%. However, this came with a significant cost advantage, bringing the total optimization cost down to roughly $2 to 4 USD per dataset. These results show that CORAL can be reproduced without relying on proprietary APIs, although there is a small drop in performance. Supporting this direction, Pozi and Sato (2025) highlight that data-augmented routing methods can achieve up to 16 × efficiency improvements in resource-constrained edge-cloud environments. To support full reproducibility, all random seeds, hyperparameter settings, and prompt evolution logs will be released along with the code.

#### 6.6.1 Computational complexity

The per-generation cost of CORAL scales as O(G × B × (N_P + N_R)), where *G* is the number of generations, *B* is the query batch size used for fitness evaluation, and N_P and N_R are the prompt and router population sizes. The dominant term is the fitness evaluation, since each candidate must be scored on the query batch. With the settings used here (*G* = 40, B = 64, N_P = 50, N_R = 30), the full run requires on the order of 2,800 model inference calls per dataset, which matches the empirical count reported above. Wall-clock runtime on a single machine with one A100 GPU for the local weak model, plus API calls for the strong model, was approximately 3.5 to 4 h per dataset. Because the populations evolve in parallel within each generation, the method is straightforward to speed up with batched inference: Increasing batch concurrency reduced the wall-clock time to under 2 h per dataset in our setup without changing the result. Importantly, this is a one-time offline cost; at deployment time, the evolved router and prompt pool add negligible overhead, since routing is a single lightweight classifier call and the prompt is a table lookup.

### 6.7 Visual summary of the approach

Figures 1, 5 summarize the two designs visually: Figure 5 shows the fixed-prompt RouteLLM pipeline and Figure 1 illustrates the CORAL architecture. The mechanism itself is described in Sections 2.1, 4.1 and is not repeated here.

**Figure 5:**
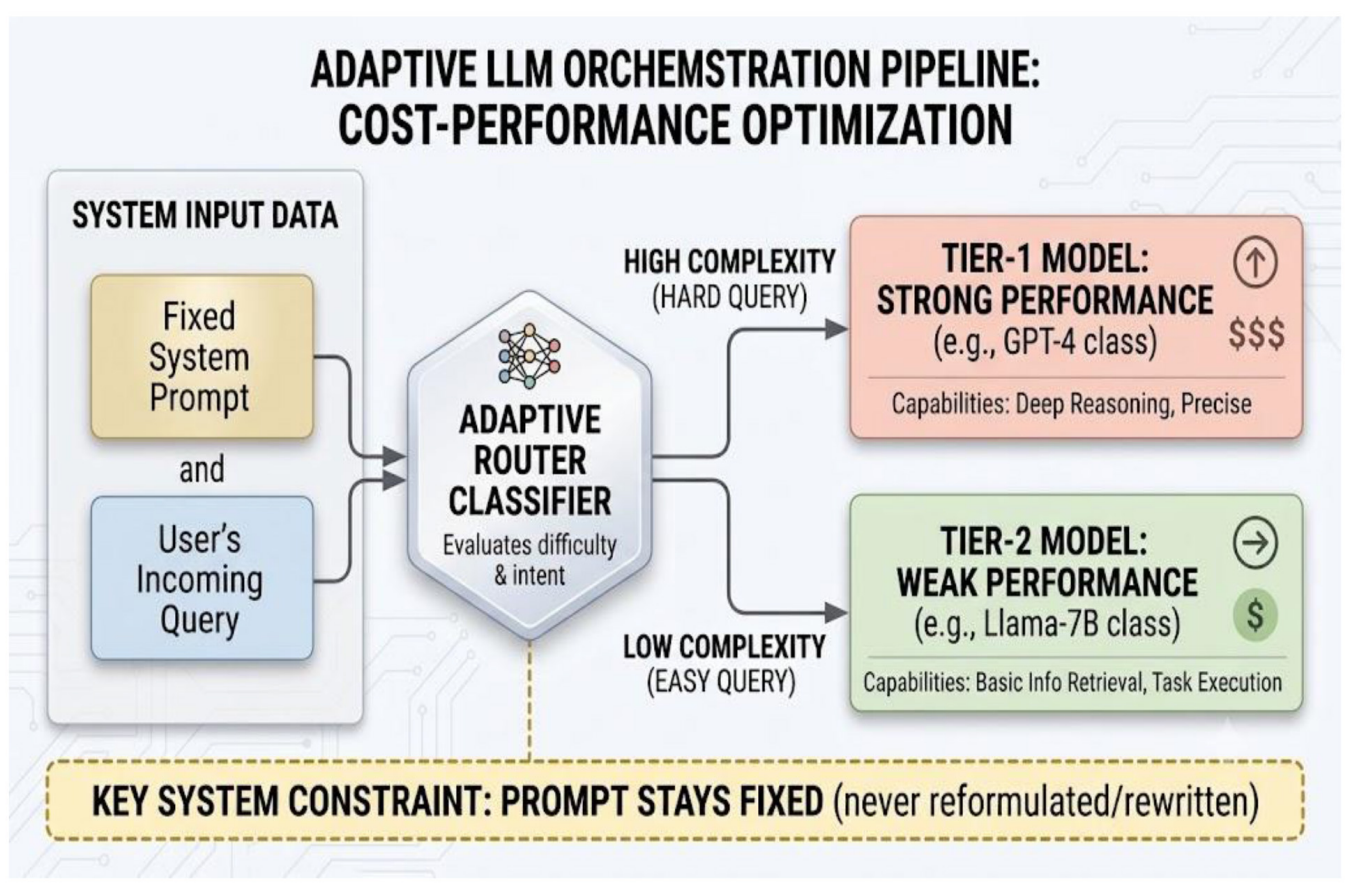
RouteLLM routes each query to a weak or strong model based on predicted difficulty, while the prompt remains unchanged.

Figure 1 shows the CORAL framework by contrast. Two populations, the prompt gene pool and the router policy pool, evolve together under multi-objective selection across accuracy, cost, and latency. The two populations exchange information through cross-population operators and a shared co-fitness evaluation, which allows the prompt and routing policy to adapt to each other rather than being fixed independently.

## 7 Discussion

### 7.1 The co-evolutionary mechanism: what is being learned

However, upon closer inspection of evolved prompt–router combinations, several intuitive patterns become evident. First, for queries destined for the weaker model on MMLU, CORAL seems to favor prompts comprising three consistent components: task instructions specifying the nature of the assignment (e.g., identifying a given question as a multiple-choice question, advising to perform a multi-step reasoning process, etc.); formatting requirements ensuring that an answer takes a certain form to minimize ambiguities; and infrequent reminders regarding domain-related facts to supplement the knowledge of the weaker model. Such optimizations clarify the task and make it easier for a less capable model, resulting in an increased number of processed queries while using fewer resources.

At the same time, prompts destined for strong models turn out to be quite concise and short, frequently shorter than the initial query. Since a strong model is capable of completing the task on its own, CORAL sees no need to include any additional information in a prompt and retains only the core part of the query. This is logical since long prompts do not benefit such models at all, making it optimal to have short, concise prompts for them. The router is modified but remains comprehensible. Over time, decision boundaries created by the classifiers within the router become more discernible and significant, aligning with the changes in prompts. By generation 40, there will be a noticeable transformation in the importance of different characteristics. For example, you will see how the router starts placing great importance on factors such as multi-step reasoning and mathematical operations performed on a given prompt, as well as complexity. This transformation reflects the process whereby the router remains aligned with the evolving prompt pool.

### 7.2 Limitations and failure modes

There are some limitations of the CORAL framework that need to be understood. First, while the offline optimization cost is relatively small compared to the savings it can bring during deployment, it is still not negligible. For applications with low traffic, specifically those handling fewer than about 10,000 queries per day, the initial optimization cost may take a long time to recover, making it less practical in such cases.

Second, CORAL's effectiveness depends on the availability of a representative training split for fitness evaluation during evolution. In zero-shot deployment settings—where no historical queries are available—the evolution cannot be initialized meaningfully. This is a fundamental limitation shared with RouteLLM and other learning-based routing approaches.

Third, the quality of LLM-mediated prompt mutation depends on the mutation LLM's ability to understand the task and generate coherent, effective prompt variants. If the mutation oracle is a weak model, the quality of evolved prompts degrades significantly. In the experiments conducted, GPT-4o was used as the mutation oracle; experiments with Llama-3.1-70B as the mutation oracle showed a 6.2% reduction in final HV, but the system remained competitive with baselines.

Fourth, CORAL currently operates in a two-model binary routing setting (weak vs. strong). Extending to multi-tier routing with *K* > 2 models increases the joint optimization space, as the router output dimensionality grows as O(|M| × |P_discrete|). Preliminary experiments with three tiers (Mistral-7B, Llama-3.1-70B, GPT-4o) showed HV = 0.1614 on MMLU vs. CORAL-full's 0.1841 (a 12.3% degradation), suggesting that CORAL's scaling to many-model settings requires architectural modifications such as hierarchical prompt–router populations. The co-evolutionary search cost scales approximately linearly with *K* for fixed population sizes, as each additional model tier adds a new class of router outputs but does not require separate populations. A full complexity analysis of PRCP for *K* > 2 models is a valuable direction for future work.

Fifth, the WildChat evaluation uses GPT-4o as a pairwise judge, introducing the risk of model self-preference bias. GPT-4o may score outputs that resemble its own generation style more favorably, regardless of content quality. While the rubric focused on factual correctness and the comparison order was randomized, this source of variance is hard to eliminate without human annotation. The WildChat cost reduction (16.3%) reported in Table 3 is less reliable than the MMLU (26.9%) and GSM8K (31.4%) results, which use exact-match scoring. Deployment decisions for open-domain query settings should treat the WildChat numbers as indicative rather than definitive.

It is equally important to state where CORAL offers little or no advantage. When almost every query in a workload is either trivially easy or genuinely hard, the band of medium-difficulty queries that prompt adaptation can shift is narrow, and the method collapses back toward plain routing; the smaller WildChat gain compared with GSM8K is an early sign of this effect. CORAL also provides little benefit when only a single model is available, since there is no routing decision to couple the prompt to, or when the strong and weak models are close in capability, because then there is little cost to save by moving queries between them. Finally, in strict low-latency settings where even a single weak-model retry is unacceptable, the occasional escalation that CORAL relies on may not be tolerable, and a fixed conservative policy may be preferred. Recognizing these cases helps practitioners avoid applying the method where its core assumption—a meaningful and adjustable difficulty gap between models—does not hold.

### 7.3 Recommendations for practitioners

The experimental findings translate into five practical recommendations for teams considering CORAL.

- First, on query volume: CORAL pays off most in settings that handle more than 10,000 queries per day, since this is the point where the one-time offline optimization cost is comfortably recovered by the per-query savings. Below this volume, the simpler fixed-prompt routing of RouteLLM may be the more sensible choice.
- Second, on task type: domains built around structured reasoning, such as mathematics, code generation, and multi-step problem solving, show the largest benefit from prompt–model coupling. Teams working in these areas should expect the biggest gains and prioritize CORAL accordingly.
- Third, on latency-sensitive deployments: where response time is tightly constrained, the NSGA-II-only configuration (CORAL-NSGAII-only) is the better fit. It reaches HV = 0.1817 against the full system's 0.1841, a negligible drop, while cutting the computational overhead of the selection step.
- Fourth, on teams without proprietary API access: The open-source Llama-3.1-70B-Instruct mutation oracle is a workable substitute for GPT-4o. It reaches HV = 0.1723 and reduces the optimization cost to roughly 2 to 4 USD per dataset, making the method reproducible on a modest budget.
- Fifth, on monitoring after deployment: Because the router and prompts are tuned on a training split, teams should periodically re-run the evolution as their live query distribution drifts, rather than treating the optimized policy as fixed for all time.

### 7.4 Comparison to DSPy and compound system optimization

CORAL is conceptually related to DSPy (Khattab et al., 2024), which compiles multimodule LLM pipelines by optimizing prompts jointly across modules. The key distinction is that DSPy targets sequential pipeline optimization (where each module feeds into the next) without explicit model routing or model selection as an optimization variable. CORAL targets routing decisions (which model to use) with prompt coadaptation, a setting that DSPy does not address. In an empirical comparison, DSPy was run with the MIPROv2 optimizer on the MMLU task using the same GPT-4o and Mistral-7B model pool, allowing DSPy to select instructions and few-shot examples. MIPROv2 achieved NACU = 0.478 at a 95% accuracy threshold on MMLU, compared to CORAL's 0.412—a 13.8% gap—confirming that DSPy's prompt optimization without co-evolutionary model routing does not recover CORAL's full Pareto improvement. Future work could explore integrating CORAL's co-evolutionary mechanism into a DSPy-like compiler that handles both pipeline sequencing and model routing jointly.

## 8 Conclusion

This study introduces CORAL, a co-evolutionary framework for the joint optimization of routing decisions and prompt generation in multi-model LLM deployment. At the heart of CORAL is a new problem formulation—the Prompt–Router Co-Evolutionary Problem (PRCP)—which captures the fundamental coupling between prompt quality and model selection that prior work has ignored. By maintaining two interacting gene pools (prompts and router policies) under NSGA-II/III multi-objective co-evolution, CORAL discovers joint (prompt, model) policies that improve on all baselines across the accuracy, cost, and latency objectives it optimizes, with the accuracy-cost trade-off summarized by the hypervolume indicator and latency reported alongside in the main results table.

Experiments on MMLU, GSM8K, and WildChat provide consistent evidence for the practical value of joint optimization: CORAL achieves up to a 31.4% cost reduction at matched accuracy and up to a 4.8 percentage-point accuracy gain at matched cost against RouteLLM on GSM8K, along with a 12.2% reduction in average per-query latency. Ablation studies confirm that the co-evolutionary coupling mechanism—not merely the addition of prompt optimization to routing—is the decisive factor in these improvements. CORAL opens several promising research directions. The extension to many-model routing with hierarchical co-evolutionary populations, the integration with streaming/online deployment where the prompt–router co-adaptation must occur continuously, the application to multi-turn conversational settings where prompt history conditions routing decisions, and the theoretical analysis of PRCP's computational complexity and approximation bounds are all rich avenues for future investigation. The main takeaway is narrower and more concrete: When the prompt is allowed to change the model a query needs, jointly searching the two decisions recovers cost-accuracy operating points that neither routing alone nor prompt tuning alone can reach.

## Data Availability

The original contributions presented in the study are included in the article/Supplementary material, further inquiries can be directed to the corresponding author.
