# Supplementary material for: CORAL: co-evolutionary optimization of routing and adaptive prompts for cost-efficient LLM deployment
Source: Front Artif Intell. 2026 Sep 15;9:1891737. doi: 10.3389/frai.2026.1891737 (PMC13619435; doi:10.3389/frai.2026.1891737)
Supplement: Supplementary file 1 [file Supplementary_file_1.docx]

**Supplementary Material**

*CORAL: co-evolutionary optimization of routing and adaptive prompts for cost-efficient LLM deployment*

Goenka S, Khekare G, Adhikari P, Tyagi A and Tyagi D

Frontiers in Artificial Intelligence | DOI 10.3389/frai.2026.1891737

**Appendix A: Dataset statistics and preprocessing**

Table A.1 provides detailed statistics for all three evaluation datasets. MMLU queries average 67 tokens in length, with a standard deviation of 41, reflecting the variable length of multiple-choice question stems and option lists. GSM8K queries are shorter on average (53 tokens) but more uniform in structure (std = 24). WildChat queries are the longest and most variable (mean = 124 tokens, std = 218), reflecting the diversity of real-world user interactions.

*Table A.1: Dataset statistics for MMLU, GSM8K, and WildChat evaluation splits.*

| **Dataset** | **Split** | **N Queries** | **Avg Tokens** | **Std Tokens** | **% Easy** | **% Hard** |
| --- | --- | --- | --- | --- | --- | --- |
| MMLU | Dev | 2,000 | 67.3 | 41.2 | 38.4% | 18.7% |
| MMLU | Test | 14,042 | 66.8 | 40.9 | 37.9% | 19.1% |
| GSM8K | Train | 7,473 | 53.1 | 24.3 | 32.1% | 22.4% |
| GSM8K | Test | 1,319 | 52.8 | 23.7 | 31.8% | 23.1% |
| WildChat | Sample | 10,000 | 124.2 | 218.1 | 29.0% | 30.0% |

**Appendix B: Prompt evolution examples**

To illustrate the prompt adaptation behaviour of CORAL, this work provides concrete examples of prompts evolved for the weak-model (Mistral-7B) tier versus the strong-model (GPT-4o) tier on GSM8K queries.

*Example: GSM8K query - ‘Natalia sold clips to 48 of her friends in April, and then she sold half as many clips in May. In April and May overall, how many clips did she sell?’*

**Original prompt:** Natalia sold clips to 48 friends in April and then sold half that many in May. How many were sold in total in both months?

**CORAL strong-model prompt:** Natalia sold clips to 48 of her friends in April, half as many in May. Total clips sold?

**CORAL weak-model prompt:**

Solve the math problem step by step and then show all the calculations

**Problem:** Natalia sold clips to 48 of her friends in April. In May, she sold exactly half as many clips as she did in April.

**Question:** How many clips did Natalia sell in total in both April and May?

Step 1: Calculate total of May sales

Step 2: Add April and May sales

Give the final answer in number

This example shows the whole co-evolution idea in CORAL. For strong models, the prompts are made simple and compressed in comparison to the original to save tokens. For weak models, the prompts are expanded with clear, step-by-step guidance that helps the model work through multi-step reasoning tasks more effectively.

Over time, the CORAL router learns to recognize patterns in the query itself. In this case, the query's structure, moderate length and straightforward arithmetic reasoning signal that it can be handled by a weak model when paired with the improved prompt. As a result, the router assigns it to the weaker and cheaper model, achieving the same outcome more efficiently.

**Appendix C: Hyperparameter sensitivity**

Table C.1 shows how sensitive CORAL's performance on MMLU (measured using the hypervolume indicator) is to different hyperparameter settings. Overall, the system remains stable across a reasonable range of configurations, including population sizes between 30 and 80 prompts (N_P = 30 to 80) and generation counts between 20 and 60 (G = 20 to 60). The mutation rate for LLM-based prompt updates has the biggest impact. If it is set too low (below 0.1), the system tends to converge too early and may miss better solutions. If it is set too high (above 0.4), the search becomes overly random, making it harder for the Pareto front to stabilize within 40 generations.

*Table C.1: Hyperparameter sensitivity on the MMLU hypervolume indicator.*

| **Parameter** | **Tested values** | **Selected value** | **Min HV** | **Max HV** |
| --- | --- | --- | --- | --- |
| N_P (prompt pool size) | 20, 30, 50, 80, 100 | 50 | 0.1643 | 0.1841 |
| N_R (router pool size) | 10, 20, 30, 50 | 30 | 0.1711 | 0.1841 |
| G (generations) | 10, 20, 40, 60 | 40 | 0.1389 | 0.1841 |
| mu_P (prompt mutation) | 0.05, 0.1, 0.2, 0.4 | 0.2 | 0.1512 | 0.1841 |
| p_xcross (cross-population) | 0.05, 0.1, 0.15, 0.25 | 0.15 | 0.1763 | 0.1841 |

**Appendix D: Latency distribution by method and dataset**

The main results table reports the mean per-query latency. Table D1 gives the full picture for every method and dataset, adding the median and the 95th-percentile (p95) latency so that the effect of tail requests is visible. All values are in milliseconds and were measured under the single-concurrency protocol with a discarded fifty-query warm-up described in the Evaluation Metrics section. As expected for language-model serving, each distribution is right-skewed: the median sits below the mean and the p95 runs well above it, driven by occasional long generations and, for the API-served strong model, variable network and load conditions.

*Table D1: Per-query latency in milliseconds for each method on each dataset, reported as mean, median, and 95th percentile (p95). Lower is better. Bold indicates the best value in each column.*

| **Method** | **Dataset** | **Mean (ms)** | **Median (ms)** | **p95 (ms)** |
| --- | --- | --- | --- | --- |
| Strong-Only | MMLU | 1840 | 1689 | 3404 |
|  | GSM8K | 1840 | 1666 | 2970 |
|  | WildChat | 1840 | 1697 | 3480 |
| Weak-Only | MMLU | 312 | 282 | 566 |
|  | GSM8K | 312 | 287 | 513 |
|  | WildChat | 312 | 286 | 515 |
| Random Routing | MMLU | 1089 | 1001 | 2008 |
|  | GSM8K | 1089 | 991 | 1860 |
|  | WildChat | 1089 | 1002 | 1922 |
| RouteLLM | MMLU | 1124 | 1034 | 1822 |
|  | GSM8K | 1124 | 1043 | 1892 |
|  | WildChat | 1124 | 1044 | 2118 |
| EvoPrompt + RouteLLM | MMLU | 1218 | 1110 | 2046 |
|  | GSM8K | 1218 | 1118 | 2147 |
|  | WildChat | 1218 | 1125 | 2310 |
| **CORAL (ours)** | **MMLU** | **987** | **896** | **1626** |
|  | **GSM8K** | **987** | **895** | **1730** |
|  | **WildChat** | **987** | **894** | **1827** |

*The mean values in Table D1 match the latency column of the main results table exactly.*
